# Imatinib Sets Pericyte Mosaic in the Retina

**DOI:** 10.3390/ijms21072522

**Published:** 2020-04-05

**Authors:** Tamás Kovács-Öller, Elena Ivanova, Gergely Szarka, Ádám J. Tengölics, Béla Völgyi, Botir T. Sagdullaev

**Affiliations:** 1 János Szentágothai Research Centre, University of Pécs, 7624 Pécs, Hungary; gergely.sz@gmail.com (G.S.); tengo.adam2@gmail.com (Á.J.T.); volgyi01@gamma.ttk.pte.hu (B.V.); 2 Retinal Electrical Synapses Research Group, National Brain Research Program (NAP 2.0), Hungarian Academy of Sciences, 1051 Budapest, Hungary; 3 Burke Neurological Institute, Department of Ophthalmology, Weill Cornell Medicine, White Plains, NY 10605, USA; eli3001@med.cornell.edu (E.I.); bos2005@med.cornell.edu (B.T.S.); 4 Medical School, University of Pécs, 7624 Pécs, Hungary; 5 Department of Experimental Zoology and Neurobiology, University of Pécs, 7624 Pécs, Hungary

**Keywords:** pericyte, retina, imatinib, Gleevec, PDGFR, neurovascular

## Abstract

The nervous system demands an adequate oxygen and metabolite exchange, making pericytes (PCs), the only vasoactive cells on the capillaries, essential to neural function. Loss of PCs is a hallmark of multiple diseases, including diabetes, Alzheimer’s, amyotrophic lateral sclerosis (ALS) and Parkinson’s. Platelet-derived growth factor receptors (PDGFRs) have been shown to be critical to PC function and survival. However, how PDGFR-mediated PC activity affects vascular homeostasis is not fully understood. Here, we tested the hypothesis that imatinib, a chemotherapeutic agent and a potent PDGFR inhibitor, alters PC distribution and thus induces vascular atrophy. We performed a morphometric analysis of the vascular elements in sham control and imatinib-treated NG2-DsRed mice. Vascular morphology and the integrity of the blood–retina barrier (BRB) were evaluated using blood albumin labeling. We found that imatinib decreased the number of PCs and blood vessel (BV) coverage in all retinal vascular layers; this was accompanied by a shrinkage of BV diameters. Surprisingly, the total length of capillaries was not altered, suggesting a preferential effect of imatinib on PCs. Furthermore, blood–retina barrier disruption was not evident. In conclusion, our data suggest that imatinib could help in treating neurovascular diseases and serve as a model for PC loss, without BRB disruption.

## 1. Introduction

Platelet-derived growth factor receptor beta (PDGFR-β) is amongst the major markers expressed by pericytes (PCs) found on the capillaries and is responsible for metabolism and oxygen supply. Together with the PDGF-BB ligand, it promotes vascular maturation and stabilization by recruiting PCs and their progenitors [1,2].

**Figure 1:**
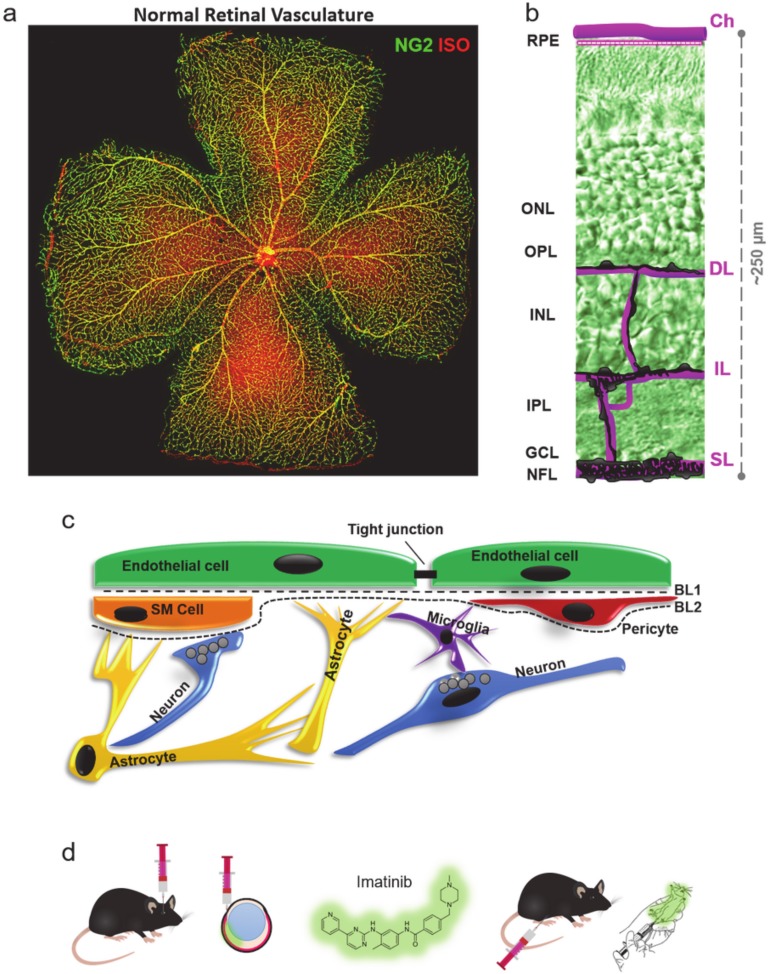
The essential role of pericytes (PCs) in the retina and in the neural tissue. (**a**) PCs (NG2—green) and retinal vasculature in the mouse retina (ISO, Isolectin GS-IB4—red). (**b**) Layers of the retinal vasculature: choroid superficial, intermediate, deep: Ch, SL, IL, DL (**c**) Location of PCs in the neurovascular unit (NVU) imatinib treatment schemes. (**d**) Local (intravitreal—IV, left) and global (intraperitoneal—IP, right) treatment with imatinib.

Although their role has been controversial for a long time [3], it is now clear that PCs are able to regulate blood flow in the nervous tissue as part of the neurovascular unit (NVU) (Figure 1c) [4,5,6,7,8]. They express alpha-smooth muscle actin, making them capable of active vasoconstriction [9], as previously functionally shown by Ivanova et al., 2017 [6]. They also have a crucial role in many diseases, such as diabetic retinopathy [6,10,11]. In PC-deficient mice, hypoxia was reported in the hippocampus, as well as inflammation, and learning and memory impairments [12]. Embryonal ablation of either PDGF-BB or PDGFR-β induces microvascular leakage and hemorrhage and is therefore lethal [13,14]. Interestingly, however, they are only essential in the formation of new blood vessels (BV) [15,16], but not in adult blood–retina barrier (BRB) function in the retina [17]. PCs regulate the blood–brain barrier (BBB) in development [17,18] while in the adult CNS, PC-deficient BVs become dilated without BBB impairment [19,20].

**Figure 2:**
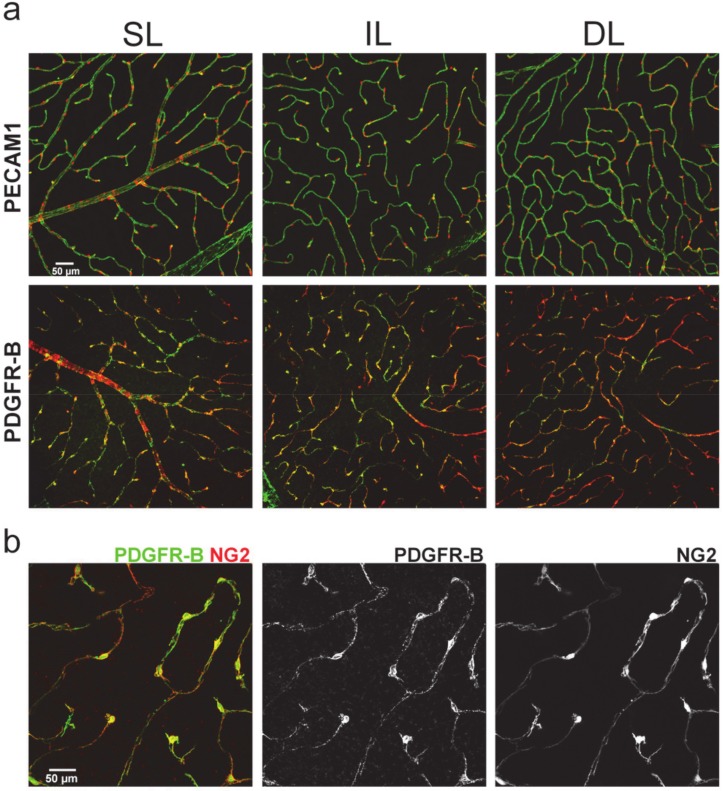
Pattern of endothelial PECAM1 (CD31) and PC PDGFR-β and NG2 in the mouse retina shows strong colocalization of PDGFR-β and NG2 in each layer of the retinal vasculature. (**a**) NG2 is strictly expressed by mural cells attached to the PECAM1+ vasculature, shown by confocal images of NG2-DsRed (red) and PECAM1/PDGFR-β (green) labeling (area 637 × 637 μm) in mouse retinas from each (superficial, intermediate, deep: SL, IL, DL) layer. PECAM1 labels endothelial cells, while (**b**) PDGFR-β is expressed by NG2+ PCs (and by smooth muscle cells, only shown on (**a**)). It recruits vascularization and stabilizes the mature vasculature. All samples were controls.

PCs express PDGFR-β while endothelial cells (EC) secrete PDGF-BB, which is an important relationship for recruiting PCs to BVs during development [21]. PDGFR-β is present on PCs, whereas mature ECs express PECAM1 (CD31) and both labels are restricted to the vasculature. In addition, PCs and smooth muscle cells (SMCs) express neural-glial marker-2 (NG2, or chondroitin sulfate proteoglycan 4 (Figure 1a). NG2 is a more suitable marker for PC morphological assessment and counting, as the somata of the cells are evenly visible and can be found in the inner retina in three separate layers [6,7,22] (Figure 1a,b and Figure 2).

Imatinib has potentially been used as a selective cancer treatment, acting through multiple signaling pathways [23,24,25]. It is a BCR-ABL tyrosine kinase inhibitor and platelet-derived growth factor receptor (PDGFR) and KIT inhibitor, and thus induces apoptosis by inhibiting PDGFs but not insulin-induced PI 3-kinase/Akt survival signaling [26]. Imatinib has also been shown to impair the migration of PCs and fibroblasts in vitro [27].

Pharmacologic intervention with PDGFR-β inhibitors, such as imatinib, has shown significant effects in attenuating angiogenesis by reducing PC density. In addition, imatinib has been shown to successfully inhibit retinal neovascularization in oxygen-induced retinopathy in development by reducing the number of vascular cells, including PCs [24,28]. However, targeting PDGFR by imatinib administration in the adult retina has not been extensively evaluated as a potential model for pathological PC loss. Here, we show a decrease of vascular areal densities, possibly as a result of PC depletion, following either local or systemic treatment of imatinib in the adult mouse retina.

In most previous studies, imatinib was injected intraperitoneally, ignoring the possible adverse effects of systemic treatment [29] (Figure 1d). However, such adverse effects, directly linking PC depletion to a decrease in BV density and retinal degeneration, have not been proven [30,31]. Optionally, a local, intravitreal treatment could be used to treat vascular diseases in the retina, as this has been shown to be most effective in many cases in treating diabetic retinopathy, AMD and retinal vein occlusion [32]. In summary, inhibition of PDGFs/PDGFRs might be a promising method for targeted PC elimination without BBB disruption. Our findings also suggest that local imatinib administration could be considered as an efficient treatment modality.

## 2. Results

The retinal vasculature supplies the retina with an adequate amount of oxygen and metabolites (Figure 1a,b), thereby proving essential for retinal survival. We utilized two treatment options, systemic, intraperitoneal injections and local, intravitreal injections of imatinib to evaluate the effect of PDGF-inhibition (Figure 1d). According to our hypothesis, imatinib treatment acts on PCs in the adult retina, and, thus, a decrease in PC number accompanied by a decrease of vascular density and interference with NVU (Figure 1c) function is expected, leading to various forms of retinal pathologies.

### 2.1. PDGFR-β Expression in the Mouse Retina Shows Colocalization with NG2

We performed PDGFR-β and PECAM1 immunohistochemical fluorescent labeling (*n* = 3 retinas) in control retinas to show their colocalization with NG2. PCs express PDGFRβ and NG2 in a colocalized manner. PECAM1 is expressed by endothelial cells, similar to normal adult retinas. We also tested our sham-control samples for any evident errors caused by PBS injection. We found no vascular aberrations in our sham control samples in PECAM1 or PDGFRβ expression (Figure 2).

### 2.2. Imatinib Decreases Blood Vessel Coverage

**Figure 3:**
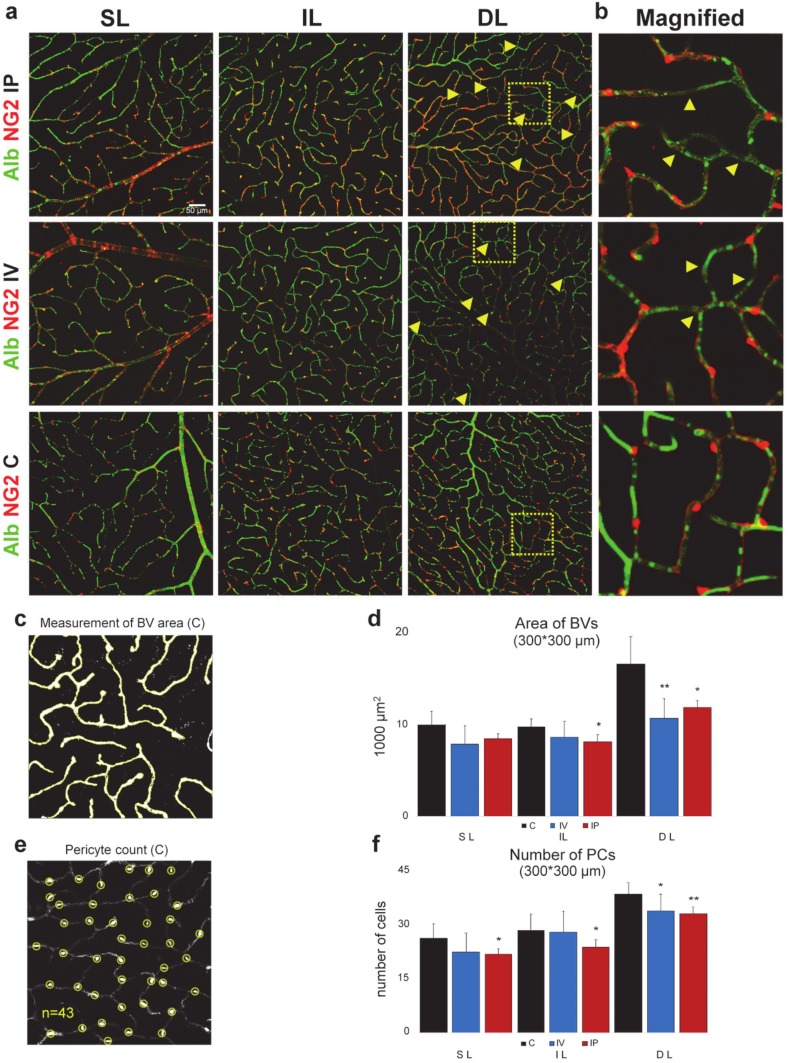
Reduction of PC count and the areal densities of blood vessels following imatinib treatment (**a**) Confocal images of NG2-DsRed (red) and Albumin (green) labeling (637 × 637 µm) in mouse retinas from each layer (superficial, intermediate, deep: SL, IL, DL). Measurements were made from these original images, layer by layer, in a 300 × 300 µm area in sham-injected, intravitreally injected (2 µL, 8 µg/µL, IV) and intraperitoneally injected (100mg/kg/d, 2 days imatinib, IP) retinas. PC loss is indicated with yellow arrows; we could not see PC loss in the sham-injected controls. Areas indicated by yellow squares are magnified and shown on panel (**b**). We found no obvious sign of any extravascular albumin label in our imatinib-treated specimen (nor our controls), suggesting that the brain–retina barrier (BRB) remained intact in retinas of these mice. *n* = 13 (C 5, IV 3, IP 5). An example for (**c**) determining BV areas and (**e**) PC numbers. (**d**) BV areas and PC numbers (**f**) showed a statistically significant decrease in the IL and DL, suggesting a non-significant decrease in the SL and in the IL of IV animals.

First, we used albumin labeling to evaluate the effect of imatinib on the vascular densities in each retinal layer. It was not evident if imatinib induced any change in the BV coverage when albumin-labeled confocal images were compared. To also obtain a quantitative comparison, we performed a measurement of areal coverage of BVs in all layers of sham controls, IV and IP treated mouse retinas. Based on this comparison, our data suggest that imatinib treatment induced a decrease in BV coverage in all layers for both treatment modalities. This decrement was evident and statistically significant in the deep layer and the intermediate layer of the IP-injected animals (tested for *n* = 4 mice for C, 3 IP, 3 IV; ANOVA; Tukey’s post hoc; *p* < 0.05) (Figure 3c,d).

### 2.3. Imatinib Treatment Decreases the Number of PCs

PCs are essential for blood vessel formation [4]; therefore, we tested if the above-observed decrease of BV areas (Figure 3d) was accompanied by a corresponding change in the number of PCs (Figure 3f) following imatinib treatment. Our data suggest that both treatment modalities resulted in a decrease in PC coverage in all three vascular layers; however, these changes were not statistically significant in the case of the SL and IL layers with IP treatment. This decrease was pronounced in all three layers of the IP-injected mice and in the deep layer in the IV-treated animals (*p* < 0.5) (Figure 3f). This means a 14.70% and 17.07% decrease in the SL; a 1.83% and 16.59% decrease in the IL; and a 12.15% and 14.01% in the DL of after IV and IP treatment respectively (Table 1). This thus means that imatinib treatment results in a significant decrease of the PC numbers in all layers in case of IP treatment, and a milder effect can be observed in the case of the local treatment in all layers (with a significant change in the DL).

To test whether there was a significant correlation between the change in PC number and the decrease in capillary surface area, using R (R Core Team 2013), we performed a Spearman’s rank correlation test (*p* < 0.01, rho = 0.7743385) and also fitted a generalized linear model to our dataset, setting the capillary layer and the treatment type as factors (PC number—Area: *p* = 0.039199). The results suggest a strong correlation between PC number and capillary area.

### 2.4. Imatinib Treatment Does Not Change the Total Length of Capillaries but Changes PC Coverage and Capillary Diameter

**Figure 4:**
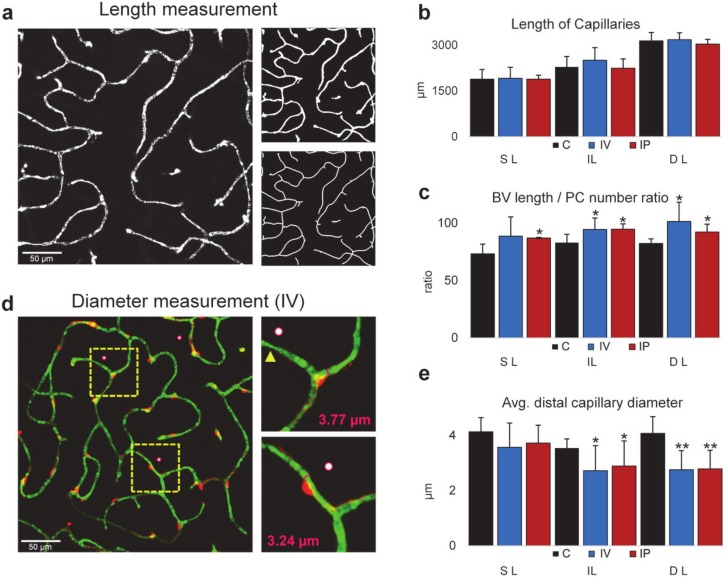
Reduction of PC number is not accompanied by BV length shrinkage following imatinib treatment. (**a**) Illustrated step-by-step method for BV length measurement: normalized grayscale image of albumin IHC labels merged from *n* = 5 optical slides (top); binary image (right top); the skeleton of BVs used to measure length (right bottom). (**b**) Comparison of blood vessel lengths in retinal layers in control (C), intravitreally injected (2 µL, 8 µg/µL imatinib, IV) and intraperitoneally injected (100 mg/kg/d, 2 days imatinib, IP) retinas. (**c**) Comparison of BV length/PC number ratios on the same dataset suggests that PCs fill out the gaps after PC loss and protect the underlying vasculature. Measurements on capillary diameters (**d**) show a reduction in the IL and DL (**e**). Measurements were made layer-by-layer in a 0.3*0.3 mm area from a mid-central region. C, IV, IP *n* = 4, 4, 3. Error bars are ± SD. * *p* < 0.05; ** *p* < 0.01.

Having proved that imatinib treatment caused PC loss (Figure 3f), the next step was to determine whether the loss of PCs had an effect on BVs. However, the total lengths in all layers remained unchanged in any of the treatment modalities (Figure 4a,b). Considering the PC-loss in both the IP- and IV-injected mice, it was not surprising that the BV length vs. PC number ratio positively changed after both of the treatments in the IL and DL and in the SL of the IP-injected animals. This change was not significant in the SL of the IV-injected mouse (Figure 4c).

However, since BV area coverage decreased while the capillary length remained constant, we had to test where the decrease in the area came from. We randomly tested capillary diameters in our dataset to show whether imatinib injection has an effect. In the case of both types of imatinib treatment modalities, capillary diameters decreased significantly in the IL and DL measured next to PC somas (Figure 4d,e), making this decrease in diameter responsible for the decrease in BV area coverage.

### 2.5. Imatinib Affected the Distribution of Capillary Branch Lengths

**Figure 5:**
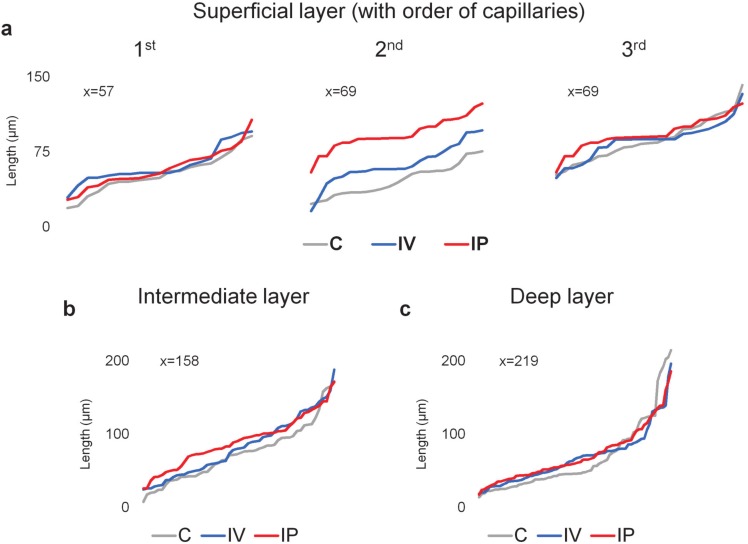
Change in the distribution of capillary branch lengths (visualized in increasing order of length within each category) in the mouse retinal vasculature after imatinib IV and IP treatments in the (**a**) SL measuring the 1st-, 2nd-, and 3rd-order capillaries (determined by the branching points after arterioles) and on capillary branches in the (**b**) IL and (**c**) DL. *n* = 6 mice; x= number of measurements. The *X*-axis represents individual length measurements in ascending order.

Imatinib induced a change in the lengths of capillary branch lengths. There was a pronounced increase after IP imatinib treatments and a milder increase in the lengths of capillaries after the second branching points in the SL (Figure 5a-2nd). We can also see a slight increase in the lower portion of capillary lengths in the IL, accompanied by no observable change in the DL (Figure 5b,c).

### 2.6. Imatinib Did Not Induce BRB Impairment

In the albumin-labeled samples, no albumin leakage into the extracellular space was detected, which would have indicated a BRB impairment (Figure 3a). This thus confirms that the BRB was intact in both sham control and imatinib-treated animals and further suggests that imatinib treatment caused milder degeneration, without BRB breach in the retinal tissue as generally observed in other degenerative processes [33].

Notably, IP-treated mice had an average 11.15% decrease (*p* = 0.065) in body weight 7 days after the first injection, suggesting a global negative effect of imatinib treatment. Nonetheless, we could not detect any characteristic change in the IV and the control animals’ weights.

## 3. Discussion

Several conditions and diseases, such as diabetes mellitus, hypertension and age-related macular degeneration (AMD) are associated with negative effects on the development and structure of the retinal vasculature [34,35]. Hypertension, for example, causes an increased contractile potential of the vessel walls and later PC loss [36]. Hyperglycemia in diabetes mellitus can also lead to microvascular diseases, including diabetic retinopathy [37].

It remains a great challenge to define the key vascular components of diabetic retinopathy; however, our present knowledge and recent discoveries in this field showed that PCs are crucial in BV formation and maintaining its structure [37]. Diabetic retinopathy is characterized by several morphological changes, including loss of PCs, thickening of the basement membrane, increased vascular permeability, vascular occlusion, and microaneurysm. Therefore, it can be concluded that PC depletion is one of the major hallmarks of diabetic retinopathy [15]. Although intraocular anti-VEGF injection and laser treatments can help regress these abnormal new vessels, they can both be detrimental or inadequate options for long-term treatment [38]. Imatinib, as a potent PDGFR, tyrosine kinase and KIT inhibitor has been successfully used in treating cancer, and thus could provide an alternative treatment strategy in neurovascular diseases. It has also been shown previously that using imatinib as a PDGFR inhibitor during retinal development after OIR (oxygen-induced retinal) neovascularization successfully ameliorated retinal degeneration in the developing retina [24]. Using genetic PDGFR-knockouts also showed PC depletion, leading to aberrant blood flow [39], albeit that such genetic modifications could be difficult in humans. In this study, we found that the PCs were significantly depleted in all three layers of the retinal BVs in the healthy, adult retina (in a fixed area, not along BVs) after IP treatment. The deep layer was the most affected, out of the three layers, in both utilized treatment options. In addition, the decreased PC number was accompanied by a reduced capillary area, and this correlation first suggested a strong association or even a causative link between these imatinib-induced effects. Even though we found no observable imatinib-induced change in the total length of BVs (Figure 4b), there was a slight local increase after the second branching point in capillary lengths in the SL and a mild increase in the lower portion of lengths in the IL, which is suggestive of minor rearrangements in the lengths, which might be related to key vascular relay PCs having a role in vasomotor response, as described before [7].

As PCs line up along capillaries, their numbers are expected to follow the length rather than the summated area of the capillary system. Our observations therefore also suggest a PC rearrangement. This hypothesis was supported by the imatinib-induced increase of the BV area/PC number ratio in both treatment modalities. This finding suggests that each retained PC covers a lengthened BV section to compensate for the imatinib-induced PC loss. This phenomenon was called pericyte structural remodeling [19,40]. While BV areas actively change in live animals, it is uncertain if the observed BV area reduction in the post-fixed state represents a corresponding BV area decrease in the live animal. Assuming that the constricted state is preserved in the fixed tissue, the detected decrease in BV areas suggests that PCs constrict after imatinib treatment. Besides the above imatinib-induced changes, layer-specific differences in PC loss were also observed (Figure 4; Table 1), which can be explained by the efficacy of local versus systemic treatment modalities. The DL is more affected, while the IL and SL is less affected by IV treatment. This may suggest that the DL is more sensitive to imatinib treatment, despite the greater distance and presumably lower local dose in IV, whereas the highest dose occurs at the level of the nearest vascular layer, the SL. On the other hand, there might be another path for IV fluid through the anterior chamber of the eye to affect the DL of retinal vasculature.

Inhibition of PDGFs/PDGFRs might be a promising therapy to suppress pathological angiogenesis. To test for imatinib-induced side effects, we measured the bodyweight of the mice before and after the treatment and labeled the retinas with albumin, to detect any BRB breakdown.

In the retina, albumin IHC has been extensively used by multiple groups to label BRB breach [33,41,42]. Armulik et al., also showed that imatinib abolishes the extravasal accumulation of intravenously injected variable-sized tracers (Evans Blue-albumin; Cadaverine-Alexa 555, MW = ~1 kDa) in the brain of PC-deficient mutants [17].

Our results show no hemorrhage and BRB breakdown after imatinib treatment either in local- or in systemic treatment tests (Figure 3), while, in parallel, the same albumin IHC adequately labels patches of extravasal albumin in the RD10 mouse retina (Figure S1). This evidence suggests that short exposure to imatinib (1 × 2 µL (8 µg/µL) IV; 2 × 100 mg/kg/day IP) already has an effect on PC density and vascular area, but not on the length or BRB integrity. This observation is consistent with other studies suggesting that PDGF-BB/PDGFR-β signaling is not required to maintain EC–PC interaction for BRB integrity during adulthood, although it is indispensable in formation and maturation of BRB through active recruitment of PCs onto the growing retinal vessels and could also be related to the suggested BRB-protective effect of imatinib [17], and elimination of PCs will eventually lead to loss of function in capillaries, thus possibly having a major effect on retinal health and resulting in further vascular atrophy.

A weight drop occurred in the IP-injected but not in the IV-injected animals after treatment, suggesting an adverse systemic effect of imatinib that can be avoided with local administration. These findings, therefore, suggest that local imatinib administration could be considered as an additional treatment modality. On the other hand, the significant weight loss that occurred after the systemic treatment points towards negative side effects of systemic imatinib administration.

In human chemotherapy, imatinib dose varies between 100–600 mg daily [43]. The highest clinical dose equals 8–12 mg/kg/day (depending on individual weight), whereas our experimental animals were treated with 100 mg/kg/day for only 2 days. Interestingly, however, this much higher dose was not disruptive of BRB integrity but decreased the PC numbers in all layers. This observation seemingly contradicts a previous study of Armulik et al. [17], where no change was detected in the BV coverage and PC numbers after imatinib treatment. However, in that study, a shorter survival time (48 h) was applied after the first treatment, whereas we waited 7 days to provide enough time for any possible rearrangement. This difference in the applied methodologies can easily explain the observed discrepancies. Additionally, local imatinib treatment may also have a somewhat different effect on retinal BV formation and maintenance. Our local IV treatment (16 µg imatinib/eye) equals a ~10-fold higher local dose in the eye compared to systemic (IP) treatment still having a less negative systemic effect, since the dosage if the IV injection is projected to the whole body weight is at least ~1500-fold lower, if we take the eye/whole body weight ratio into account.

If used for a longer period, imatinib could cause retinal pathologies in chemotherapy [30,31,44]. This long-term imatinib effect on vasculature may be explained by the loss of the PC dynamic interactions and thus the ability to sustain retinal metabolic needs in some conditions [6].

Additionally, undeniably, imatinib treatment is not specific for PC elimination, since it is not exclusively specific for PDGFR-β; it could serve as a good model for PC dropout, having a role in multiple diseases. To focus its effect and to avoid possible side effects, our results suggest a local treatment on BVs if possible. Since imatinib has been approved for medical use, there are fewer obstacles ahead to benefits from its effect.

Finally, as we have recently shown, PCs form an essential 3D mosaic across the retina to provide a propagating vasomotor response in capillaries [22]. The signal for this propagation travels across gap junctions between neighboring PCs, thereby precisely supporting metabolic needs in the nervous tissue [6,7,22]. Any loss in this 3D PC structure contributes to the loss of this essential function. We theorize that the PC mosaic can adapt to such changes to restore the function by filling out the gaps along the vasculature, which primarily resulted from the loss of some PCs. The given PC loss, without a negative change in BV length, suggests that PCs may extend their processes and even move to rebuild the mosaic in order to protect BVs from degeneration. The decrease in capillary areas may come from either a decrease in their length or a decrease in their diameter. However different labels could give slightly different diameter measurements (PECAM1) [45]. Our results were made using only albumin labeling, following the inner lumen of the capillaries. We found that the diameters indeed shrunk after treatments. Although this conflicts with other findings [17], it could be explained by the longer recovery time (7 days). After our treatments, stress might be still present, caused by PC mosaic reorganization. Although this theory needs to be further tested by directly showing apoptosis in the NVU after multiple time points, it also serves as a direct explanation and a future path for PC research.

## 4. Materials and Methods

### 4.1. Animals and Imatinib Treatment

Animal handling, housing, and experimental procedures were reviewed and approved by the ethical committee of the University of Pécs (BA02/2000-6/2006; BA/35/51-42/2016, approved on 14 April 2016) and in compliance with protocols approved by the Institutional Animal Care and Use Committee of Weill Cornell Medicine. All animals were treated in accordance with the ARVO Statement for the Use of Animals in Ophthalmic and Vision Research and with the National Institutes of Health Guide for the care and use of laboratory animals. All efforts were made to minimize pain and discomfort during the experiments and all procedures were done by obeying the 3R law.

We used multiple routes of imatinib administration to evaluate the effects of global and local use of imatinib. Three mouse lines: C57BL/6J mice (The Jackson Laboratory, stock #000664, RRID: IMSR_JAX:000664), NG2-DsRed mice (The Jackson Laboratory, Tg(Cspg4-DsRed.T1)1Akik/J, stock #008241, RRID: IMSR_JAX:008241) and RD10 (The Jackson Laboratory, stock #004297, RRID: MGI_3581193, see only in Figure S1.) were used in this study. All animals were healthy, adult (1–6 mo.), males. Animals were injected either with imatinib (Methanesulfonate Salt, LC Laboratories, Cat. I-5508) or filtered phosphate-buffered saline (PBS; 0.1 M, pH = 7.3) as follows:

- Imatinib intraperitoneal injection (IP) 100 mg/kg/day imatinib (dosage according to Raimondi 2014) in filtered PBS for 2 days, on *n* = 5 mice.
- Imatinib intravitreal injection (IV) 2 µl (8µg/µl) in filtered PBS on *n* = 3 mice.
- A/B equal volume injected from filtered PBS both IP and IV on *n* = 5 mice; used as a sham control.

### 4.2. Immunohistochemistry

Eyecups of mice were fixed with 4% carbodiimide and 0.25% PFA in 0.1 M phosphate saline (PBS, pH 7.3) for 15 min at room temperature. After fixation, the samples were washed in PBS, and the retinas were dissected. Retinal whole mounts were blocked for 8–14 h in CTA (PBS solution containing 5% Chemiblocker (membrane-blocking agent, Millipore), 0.5% Triton X-100, and 0.05% sodium azide, Sigma). Primary antibodies were diluted in the same solution and applied for 72 h, followed by incubation for 48 h in the appropriate secondary antibody, conjugated to Alexa-488 (1:1000; green fluorescence, Invitrogen), Alexa-568 (1:1000; red fluorescence, Invitrogen), Alexa-633 (1:500; far-red fluorescence, Invitrogen). In multi-labeling experiments, whole mounts were incubated in a mixture of primary antibodies, followed by a mixture of secondary antibodies. All steps were performed at room temperature. After staining, the retinal pieces were flat-mounted on a slide, ganglion cell layer up, and coverslipped using Vectashield mounting medium (H-1000, Vector Laboratories). The coverslip was sealed with nail polish using small pieces of a broken glass coverslip (number 1 size) as spacers. The primary antibodies used in this study were the following: rabbit anti-NG2 coupled to Cy3 fluorescent label (NG2, 1:500, EMD Millipore, AB5320C3), goat anti-mouse albumin (albumin, 1:1000, Bethyl Laboratories, A90-234A), goat anti-mouse PECAM1 (alternatively CD31; 1:5000, R&D Systems, AF3628) and goat anti-mouse PDGFR-β (1:5000, R&D Systems, AF1042). BVs were visualized by Isolectin coupled to Alexa-488 fluorescent label (1:500, Invitrogen, I21411) to show the whole retinal vasculature with secondary antibodies (Figure 1). Retinal samples were imaged under a Nikon Eclipse Ti-U confocal microscope. For PC evaluation, retinas were imaged with 20× objective and areas of 300 × 300 μm^2^ were selected for measurements (5 IP animals, 10 areas; 3 IV animals, 6 areas; 5 control animals, 8 areas). The samples were imaged under identical acquisition conditions, including laser intensity, photomultiplier amplification, and Z-stack step size.

### 4.3. Image Analysis

All images were processed and analyzed using FIJI software (ImageJ, NIH; [46]). For measurements, we used areas of 300 × 300 μm^2^, merged (from 5–8 optical slices) images. BVs, labeled by albumin, were traced with thresholding in FIJI and their total area was measured. NG2-positive PC numbers were counted along capillaries, considering the local BV diameter and cell morphology, using the cell counter plugin in FIJI. In total, we evaluated 1565 individual PCs from all sample sets.

Albumin labeling was also used to evaluate BV length. After initial thresholding and global processing, images were converted to binary black and white. We used the dilate and erode binary function subsequently to smooth local unevenness at BV edges. Skeletonized BVs were measured using the analyze skeleton function in FIJI. Results were transferred to Excel for further processing.

Distal capillaries were specified by counting the 2nd or 3rd bifurcation from the arteriole and considering the diameter of BV and PC morphology (bump on the log) specific for distal capillaries (Figure 4). The diameter evaluation was made on composite images. First, a custom-made ImageJ script was used to generate random points on the images. We measured the diameter with the FIJI line tool on the nearest capillary from this point, next to the PC soma (Figure 4d).

We performed capillary-branch length measurements in the SL, measuring the 1st-, 2nd-, and 3rd-order capillaries after arterioles and on capillary branches in the IL and DL. The capillary lengths were sorted from shortest to longest to show their continuous distribution in Figure 5.

### 4.4. Statistical Analysis

For statistical analysis, Excel (Microsoft Corp., Redmond, USA), JASP (JASP Team, 2019 JASP, V 0.11.1), and R (R Core Team 2013) were used. An independent t-test was used to determine the BV/PC number ratio significance. For multiple comparisons, analysis of variance (ANOVA) with post-hoc Tukey’s test or repeated-measures ANOVA was used with Pearson’s correlation matrix. The number of samples (*n*) indicates the number of mice in the group or else is indicated otherwise locally. The data is presented as mean ± SD unless otherwise indicated.

## Figures and Tables

**Table 1:** Layer-specific PC counts in the experimental specimens.

PC Numbers					
Treatment	Position	Mean	SD	Dev. from Control (%)	N
C	DL	38.375	3.114		8
	IL	28.375	4.502		8
	SL	26.125	3.980		8
IP	DL	33.000	1.732	−14.01	3
	IL	23.667	2.082	−16.59	3
	SL	21.667	1.528	−17.07	3
IV	DL	33.714	4.572	−12.15	7
	IL	27.857	5.669	−1.83	7
	SL	22.286	5.251	−14.70	7

C: sham-treated control, IP: intraperitoneally and IV: intravitreally imatinib-treated.

## Supplementary Materials

Supplementary materials can be found at https://www.mdpi.com/1422-0067/21/7/2522/s1. Figure S1. Albumin detection in the BRB disrupted RD10 mouse retina using the same IHC-method serves as a positive control for albumin extravasation (encircled by yellow, dashed lines) not detectable in our sampleset. Alb—albumin; NG2—Neural glial factor-2; ISO—Isolectin (from Griffonia simplicifolia-IB4).

## Abbreviations

ALS	Amyotrophic lateral sclerosis
AMD	Age-related macular degeneration
BBB	Blood-brain barrier
BRB	Blood–retina barrier
BV	Blood Vessel
C	Control
Ch	Choroid
CNS	Central nervous system
Cspg4	Chondroitin sulfate proteoglycan 4
DL	Deep Layer
EC	Endothelial cell
GCL	Ganglion cell layer
GS-IB4	Griffonia simplicifolia - IB4
IHC	Immunohistochemistry
IL	Intermediate Layer
INL	Inner nuclear layer
IP	Intraperitoneal
IPL	Inner plexiform layer
IV	Intravitreal
NFL	Nerve fiber layer
NG2	Neuron-glial antigen 2
NVU	Neurovascular Unit
OIR	oxygen-induced retinal
ONL	Outer nuclear layer
OPL	Outer plexiform layer
PBS	Phosphate-buffered saline
PC	Pericytes
PDGF	Platelet-derived growth factor
PDGFR	Platelet-derived growth factor receptor
PDGFR-β	Platelet-Derived Growth Factor Receptor-Beta
PECAM1	Platelet and endothelial cell adhesion molecule 1
RPE	Retinal pigment epithelium
SD	Standard deviation
SL	Superficial Layer
SMC	Smooth muscle cells
VEGF	Vascular endothelial growth factor
