# Supplementary figures and images for: Imatinib Sets Pericyte Mosaic in the Retina
Source: Int J Mol Sci. 2020 Apr 5;21(7):2522. doi: 10.3390/ijms21072522 (PMC7177598; doi:10.3390/ijms21072522)

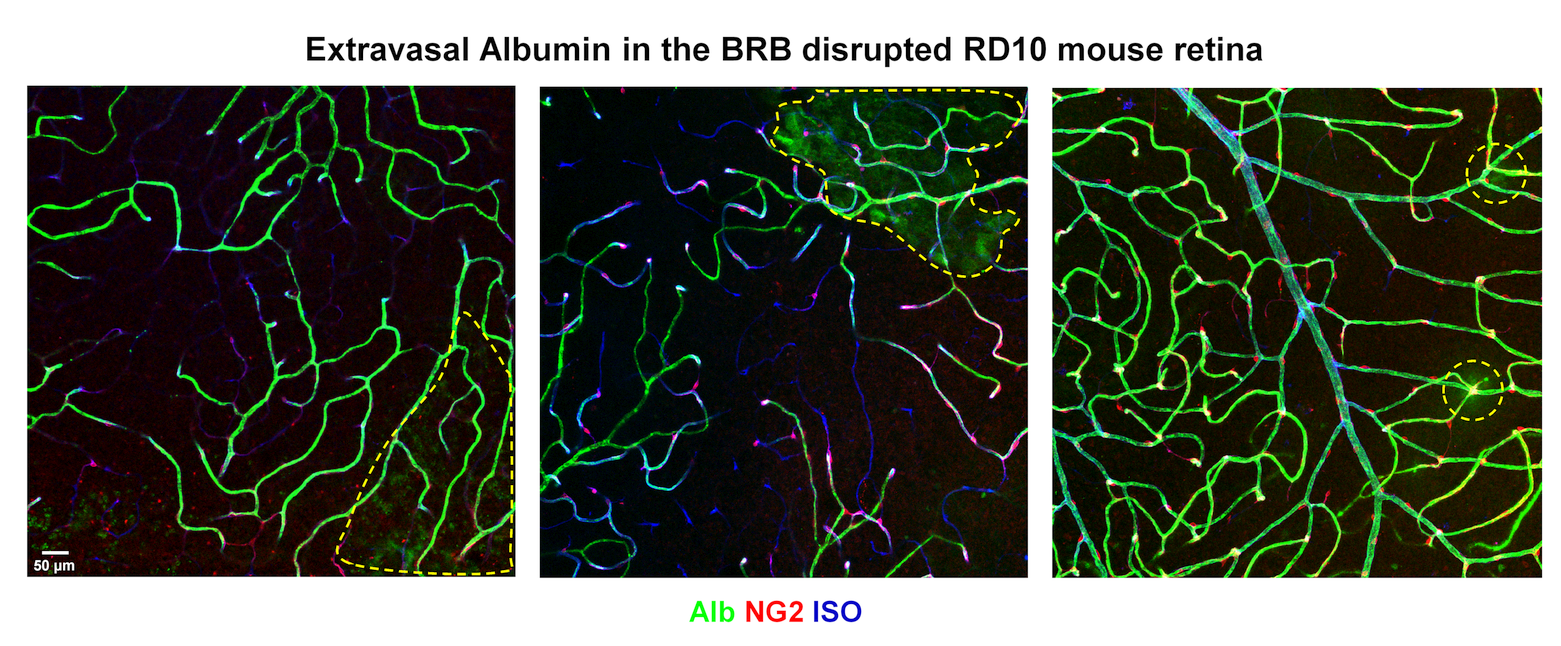

Supplement: Supplementary file 1 [file ijms-21-02522-s001.zip › SupF1.tif]
